# The distribution of serotonergic nerve on the hippocampus of the fruit bats (*Rousettus amplexicaudatus*)

**DOI:** 10.14202/vetworld.2019.1460-1466

**Published:** 2019-09

**Authors:** Vivin Wirawati, Nourrisma D. A. Widiati, Geraldus Gunawan, Golda R. Saragih, Puspa Hening, Hevi Wihadmadyatami

**Affiliations:** 1 Department of Anatomy, Faculty of Veterinary Medicine, Universitas Gadjah Mada, Yogyakarta 55281, Indonesia; 2 Integrated Laboratory for Research and Testing, Universitas Gadjah Mada, Yogyakarta 55281, Indonesia

**Keywords:** hippocampus, immunohistochemistry, learning and memory, *Rousettus amplexicaudatus*, serotonin

## Abstract

**Background:**

*Rousettus amplexicaudatus* is one of the old world fruit bats, commonly known in Javanese word as codot belongs to Order Chiroptera and suborder Megachiroptera. *R. amplexicaudatus* possessed an outstanding visual ability, which helps in the process of searching for food. Those activities process and save within the brain involving the learning and memory activities. Learning and memory activities are centered around the hippocampus with the help of serotonergic nerve.

**Aim:**

This study conducted to identify the morphology and distribution of serotonergic nerve (5-hydroxytryptamine) in the hippocampus of *R. amplexicaudatus*, which related to the function of serotonin within the learning and memory process.

**Materials and Methods:**

Five *R. amplexicaudatus* bats were brought from Gunung Kidul cave, Yogyakarta, Java Island, Indonesia. The bats were anesthetized using ketamine and xylazine. *R. amplexicaudatus* then perfused intracardially. The brain samples were collected processed into paraffin block, and a further cut in 8 µm thickness serially. The tissue slides were stained using hematoxylin-eosin, cresyl echt violet, and immunohistochemistry with rabbit’s anti-serotonin (1:200). The results observed and analyzed quantitatively and qualitatively using image J (https://imagej.nih.gov/ij/) (Bethesda, Maryland, USA) and GraphPad Prism 7 (La Jolla, CA, USA).

**Results:**

The hippocampus of *R. amplexicaudatus* composed of the dentate gyrus (DG), hippocampus proper (cornu ammonis 3 [CA3], and CA1 as the main area for learning memory), and subiculum. On the DG serotonin immunoreactive cells found within the granular layer (132±35.03 cells/mm^2^), polymorphic stratum (86.33±11.23 cells/mm^2^), and molecular layer (93±1 cells/mm^2^). Meanwhile, on CA3 area, the immunoreactive cells for serotonin found in each stratum. The number of immunoreactive cells on each stratum from highest to the lowest are stratum pyramidal 123.33±15.88 cell/mm^2^, stratum molecular 63±13.11 cell/mm^2^, stratum lucidum 62.67±8.08 cell/mm^2^, stratum radiatum 55.33±510.21 cell/mm^2^, stratum oriens 48±3.46 cell/mm^2^, and stratum alveus 28.67±2,52 cell/mm^2^. In addition, in CA1 also hampers the immunoreactive cells in the pyramidal stratum, molecular, lucidum, oriens, radiatum, and alveus layer, respectively, of each 91±27.40 cell/mm^2^, 60.33±20.65 cell/mm^2^, 53.67±4.51 cell/mm^2^44.33±10.40 cell/mm^2^, 41.33±5.51 cell/mm^2^, and 27±4 cell/mm^2^.

**Conclusion:**

Taking together the distribution of serotonin-immunoreactive cells in the hippocampus of *R. amplexicaudatus* mostly found on CA3 followed by CA 1 and DG.

## Introduction

Indonesia is a country with a large amount of bat biodiversity. In Indonesia already found 241 species of bat or around 21% from total species which are present in the world. The bats in Indonesia consist of ten families, 78 species of frugivore (Megachiroptera) and 153 species of insectivore (Microchiroptera) [1]. Those ten families are *Hipposideridae, Emballonuridae, Vespertilionidae, Pteropodidae, Megadermatidae, Nycteridae*, Rhinopomatidae, Miniopteridae, *Molossidae*, and Rhinolophidae [2].

The *Rousettus amplexicaudatus* is one of the frugivores, mainly in central Java and special region of Yogyakarta, Indonesia, known as codot. *R. amplexicaudatus* belongs to suborder Megachiroptera or megabats and has a big size with high visualization ability, great learning, and memory activities which centered in the hippocampus. *R. amplexicaudatus* is a nocturnal animal; it searches for foods at night and relies on its excellent visualization ability [3]. This activity will be recorded by central nervous system as a center of learning and memory activities [4]. Learning and memory processes in bats are carried out by the limbic system, which consists of limbic cortex, hippocampus, amygdala, septal area, and hypothalamus that support each other [5]. It is also mentioned that learning and memory activities happen in the hippocampus of mammals and invertebrate animals [4]. Learning and memory in the hippocampus are affected by the presence of signals from neuromodulatory transmitter action. One of neuromodulator that takes the role in learning and memory is serotonin (5-hydroxytryptamine [5-HT]) [4].

Serotonin (5-HT) is a neurotransmitter which produced from essential amino acid and tryptophan through two-step synthetic pathway. Serotonin is monoamine neurotransmitter that has chemical structure from essential elements of the amino acid group, which is divided into an aromatic nucleus by two aliphatic carbon chains [6]. Some research already describes that serotonin is found on Cornu ammonis 1 (CA1) and CA3 in the hippocampus, which is associated with cognitive, learning activities, and memory formation [4]. In addition, *R. amplexicaudatus* has promoted both sensory and behavioral adaptations that allow them to find ripe fruit faster, but the exact mechanism and the neurotransmitters involved in this process are still not widely known.

This study aimed to describe for the 1^st^ time the distribution of serotonergic nerve on the hippocampus of the *R. amplexicaudatus* and explain the possible mechanism of serotonin in the cognitive and also memory formation based on literature study.

## Materials and Methods

### Ethical approval

The whole process of this research was approved by the Ethics Committee of the Faculty of Veterinary Medicine, Universitas Gadjah Mada, with the number 0045/EC-FKH/Int/2019.

### Animals

Five fruit bats (*R. amplexicaudatus*) which is classified under the International Union for Conservation of Natures Least Concern category were anesthetized with ketamine (Kepro, Maagdenburgstraat, Holland) (10 mg/kg body weight) and xylazine (Interchemie, Metaalweg, Holland) (2 mg/kg body weight) which were injected intramuscularly. Animals were perfused intracardially using NaCl (Nacalai Tesque, Kyoto, Japan) as pre-rinse. The thorax cavity was opened by cutting the lateral side of os costa. After cavum thorax was opened and cor was seen, then it was perfused intracardially using physiological NaCl solution through the ventricular sinister. As NaCl entered the cor, auricular atrium dexter was opened by cutting to bring out the blood. After the blood was successfully removed, the solution was replaced by 4% formaldehyde (Nacalai Tesque, Kyoto, Japan) as a fixative solution. After all, tissues were fixed, brains were processed and stored in formaldehyde 4% as a fixative solution.

### Tissue embedding in liquid paraffin

The first step of tissue processing in the paraffin method was dehydration. It was performed by immersing the tissues on a series of alcohol (KgaA, Darmstadt, Germany) for 60 min each. The second step was clearing step; it was performed by soaking the tissues into a series of xylol (KgaA, Darmstadt, Germany) 60 min each. The third step was paraffin infiltration; paraffins I, II, and III were used at 60 min of incubation in an incubator at 58-60°C. The last step of this tissue processing was embedding and blocking in paraffin. Blocking was performed on the heating furnace; then, the liquid paraffin (Leica Biosystems, Richmond, USA) was poured into the mold on the heating furnace. The tissue was placed on the paraffin in a transverse position, and then after the paraffin freezes, it was stored in the refrigerator. The frozen paraffin block was then cut using a rotary microtome (Yamato RV 240, Asaka, Japan) of 8-µm thickness. The section was placed on the surface of cold water and then fixed to the gelatin-coated object-glass. The slides were incubated and put on the hotplate at ±40°C for 1 h.

### Immunohistochemistry staining of serotonin

The slides were soaked in graded series of xylol, rehydrated by soaking on a series of alcohol and then washed with aquadest 3 times for 3 min. The tissues were put on a low-temperature microwave for 10 min for antigen retrieval. Slides were put on H_2_O_2_ 3% in methanol (9 ml H_2_O_2_ dissolved in 81 ml absolute methanol) for 15 min at room temperature. The slides were washed with phosphate-buffered saline (PBS) and dripped with Biocare Background Sniper, incubated for 15 min at room temperature and placed horizontally in closed and moist box. 30 µl primary antibody anti-serotonin rabbit serum (1:200) was dripped in each of tissue incision. Slide was closed with parafilm and incubated for 48 h at 4°C. The tissues were washed with PBS then dripped with secondary antibody Trekkie Universal Link and incubated for 20 min. After washed with PBS, the tissues were incubated in Trek-Avidin Horseradish Peroxidase Label for 5 min at room temperature. 30 µl dimethylaminobenzaldehyde (DAB) dripped on the tissues. The tissues were dehydrated using a series of alcohol, clearing using a series of xylol and ended with mounting using Canada balsam.

### Hematoxylin-eosin staining

The slides were deparaffinized by soaking the tissues in a graded series of xylol for 5 min. The slides were then rehydrated in the graded series of absolute alcohol for 3 min. The tissue sections were stained with hematoxylin and eosin (Bio Optica, Milano, Spanyol), observed under a light microscope (Nikon, Tokyo, Japan). Pictures were taken using software Optilab (Optilab, Yogyakarta, Indonesia).

### Cresyl echt violet staining

Cresyl echt violet stain was incubated at 40°C for 24 h before staining, while the tissue slide was incubated at 40°C for 1 h before staining. Deparaffinization was performed using xylol for 5 min, rehydrated using graded series of alcohol for 5 min then soaked into cresyl echt violet at 37.5°C for 30 min and aquadest for 1 min. The slide was dehydrated using a graded series of alcohol for 15 s, cleared by xylol solution for 5 min then cover with deck glass using Canada balsam.

### Statistical analysis

The cell numbers were analyzed quantitatively and qualitatively using an image J program from https://imagej.nih.gov/ij/(Bethesda, Maryland, USA). Statistical analysis was made using descriptive statistics to calculate the average number of cells and the standard deviation. Statistics were performed using GraphPad Prism 7 (La Jolla, CA, USA).

## Results

### Histological structure of the hippocampal formation in fruit bats

**Figure-1:**
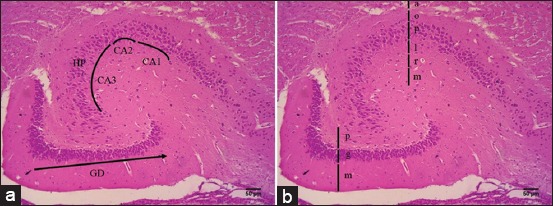
The histological structure of hippocampus *Rousettus amplexicaudatus* in the hematoxylin and eosin staining. (a) Region of hippocampus consists of the dentate gyrus (GD), hippocampus proper (HC) (Cornu ammonis 1 [CA1], CA2, and CA3), and subiculum (S). (b) The layer of DG and hippocampus proper of *R. amplexicaudatus*. DG consists of stratum molecular (m), stratum granular (g), stratum polymorphic (p). Meanwhile, hippocampus proper consists of stratum alveolus (a), stratum oriens (o), stratum pyramidal (p), stratum lucidum (l), stratum radiatum (r), stratum molecular (m).

**Figure-2:**
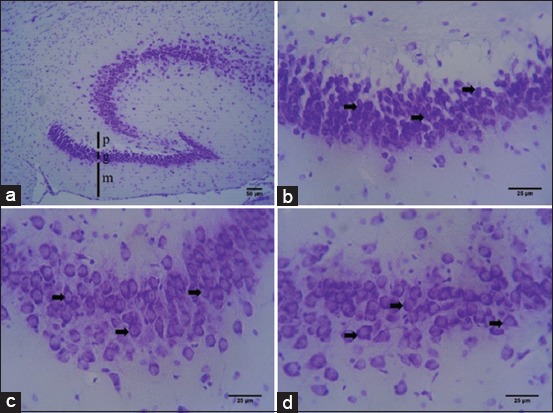
The histological structure of *Rousettus amplexicaudatus* hippocampus with Cresyl echt violet staining. (a) The dentate gyrus (DG) area differentiates becomes three-layer: Stratum polymorphic (p), stratum granular (g), stratum molecular. (b) The stratum granular (g) of DG composed of granular cells (black arrow) (40×). (c) Stratum pyramidale of CA3, consist of pyramidal cells in high density (black arrow). (d) Stratum pyramidale of CA1, consist of pyramidal cells in low density (black arrow).

Based on hematoxylin-eosin (Figure-1) and cresyl echt violet staining (Figure-2), we are able to distinguish and identify the histological structure of the hippocampal formation in fruit bats. It identified three regions of *Rousettus*’ hippocampus, consisting of dentate gyrus (DG), and hippocampus proper and subiculum (Figure-1a). Hippocampus proper is divided into CA1, CA2, and CA3 (Figure-1a). Hippocampus proper of *R. amplexicaudatus* has six stratums from superficial to profundus; there are stratum alveus, stratum oriens, stratum pyramidal, stratum lucidum, stratum radiatum, and stratum molecular (Figure-1b). Stratum pyramidal of CA3 consists of pyramidal cells with high density (Figure-2c) meanwhile in CA1 consists of pyramidal cells with low density (Figure-2d). DG of *R. amplexicaudatus* consists of stratum molecular (Figures-1b and 2a), stratum granular (Figures-1b, 2a and b), and stratum polymorphic (Figures-1b and 2a).

### Distribution of serotonin on hippocampus area of *R. amplexicaudatus*

**Figure-3:**
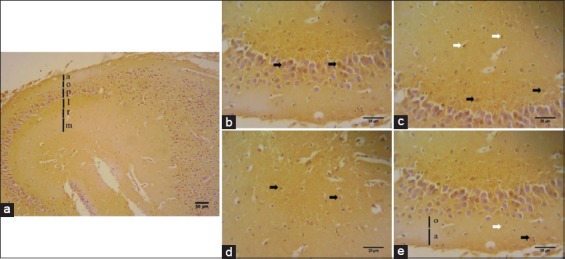
The immunohistochemistry of serotonin on the Cornu ammonis 1 (CA1) (a) the CA1 of *Rousettus amplexicaudatus* divided into six layers: (a) stratum alveus, (o) stratum oriens, (p) stratum pyramidale, (l) stratum lucidum, (r) stratum radiatum, and (m) stratum molecular. The serotonin immunoreactive cells found on (b) stratum pyramidal (black arrow), (c) stratum lucidum (black arrow) and stratum radiatum (white arrow), (d) stratum molecular (black arrow), and (e) stratum oriens (white arrow) and stratum alveus (black arrow).

**Figure-4:**
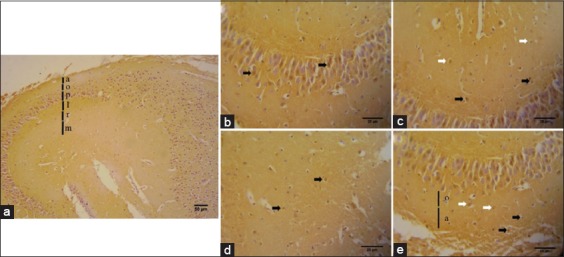
The immunohistochemistry of serotonin on the Cornu ammonis 3 (CA3) (a) The CA3 of *Rousettus amplexicaudatus* differentiate in six stratum: (a) Stratum alveus, (o) stratum oriens, (p) stratum pyramidale, (l) stratum lucidum, (r) stratum radiatum, (m) stratum molecular. Serotonin immunoreactive cells distribute on (b) stratum pyramidal (black arrow), (c) stratum lucidum (black arrow) and stratum radiatum (white arrow), (d) stratum molecular (black arrow), and (e) stratum oriens (white arrow) and stratum alveus (black arrow).

**Figure-5:**
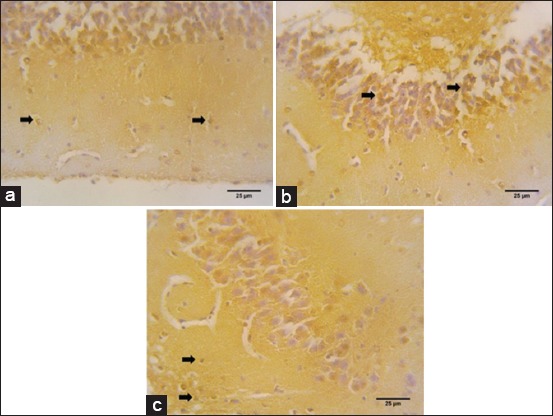
Serotonin distribution on the dentate gyrus of *Rousettus amplexicaudatus* in immunohistochemistry staining. Serotonin immunoreactive cells are found on (a) stratum molecular (black arrow), (b) stratum granular (black arrow), and (c) stratum polymorphic (black arrow).

**Figure-6:**
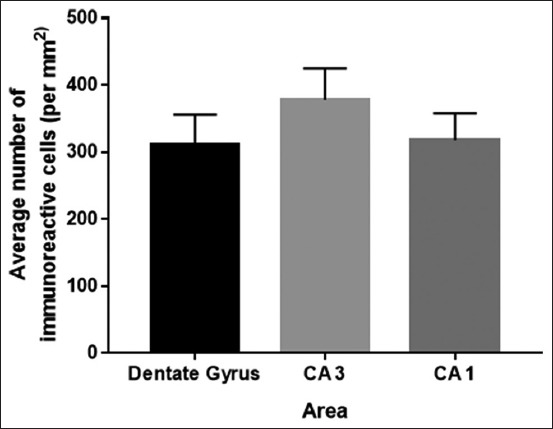
The distribution serotonin immunoreactive in the hippocampus of *Rousettus amplexicaudatus* is mostly found on Cornu ammonis 3 (CA3), followed by CA1 area and dentate gyrus.

Serotonin immunohistochemistry aims to detect the location, distribution, and expression of serotonin on hippocampus area of *R. amplexicaudatus*. The results obtained from immunohistochemical staining using serotonin antibodies are cells in the DG, CA1, and CA3 areas are immunoreactive against anti-serotonin (Figures-3-5). The visible color is brown from DAB chromogen and purple from Harris hematoxylin counterstain, which stains the nucleus. Cell bodies which were immunoreactive to serotonin are observed on CA1, CA3, and DG area. The number of serotonin immunoreactive cells from highest to lowest is CA3 (378±46.81 cells/mm^2^), CA1 (317.68±40.28 cells/mm^2^), and DG (311.33±44.66 cells/mm^2^) (Figure-6).

We also identify serotonin immunoreactive cells on CA3 and CA1. Immunoreactive cells against anti-serotonin can be found in CA1’s pyramidal cells (Figure-3b), CA3’s pyramidal cells (Figure-4b), and DG’ granular cells (Figure-5b).

### Serotonin immunoreactive cells on CA1

**Figure-7:**
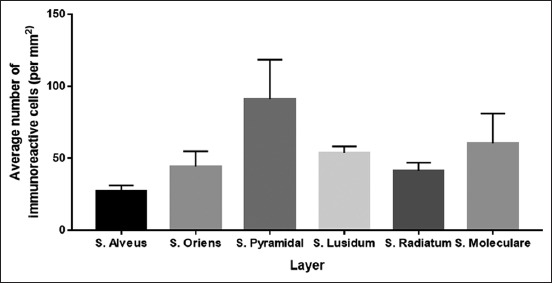
The number of immunoreactive cells from the highest to lowest in Cornu ammonis 1. The highest is stratum pyramidal followed by stratum molecular, stratum lucidum, stratum oriens, stratum radiatum, and stratum alveus.

Serotonin immunohistochemistry staining on the hippocampus of *R. amplexicaudatus* result shows immunoreactive cells in each of CA1 layer (Figure-3a). Immunoreactive cells are mainly found in pyramidal cells of stratum pyramidal (Figure-3b), stratum lucidum containing mossy fiber (Figure-3c), and interneuron of stratum molecular (Figure-3d). Calculation of immunoreactive cells on each stratum of CA1 result in the three layers with the highest immunoreactivity levels, namely, stratum pyramidal, stratum molecular, and stratum lucidum. The average number of cells and their deviation standards is stratum pyramidal (91±27.40 cells/mm^2^), stratum molecular (60.33±20.65 cells/mm^2^), stratum lucidum (53.67±4.51 cells/mm^2^), stratum oriens (53.67±4.51 cells/mm^2^), stratum radiatum (41.33±5.51 cells/mm^2^), and stratum alveus (27±4 cells/mm^2^) (Figure-7).

### Serotonin immunoreactive cells on CA3

**Figure-8:**
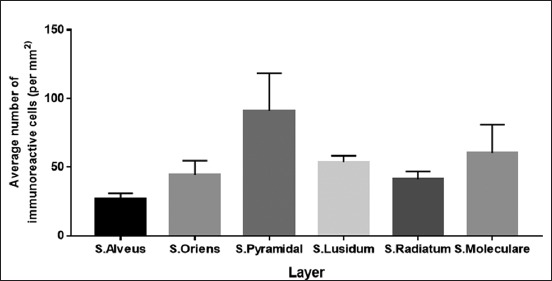
The number of immunoreactive cells from the highest to lowest in CA3 is stratum pyramidal, stratum molecular, stratum lucidum, stratum oriens, stratum radiatum, and stratum alveus.

Serotonin immunohistochemistry staining on *R. amplexicaudatus*’ hippocampus shows immunoreactive cells in each layer (Figure-4a). Immunoreactive cells are mainly found in pyramidal cells of stratum pyramidal (Figure-4b), stratum lucidum containing mossy fiber (Figure-4c), stratum molecular which containing interneuron cells (Figure-4d), stratum oriens, and stratum alveus (Figure-4e). Calculation of immunoreactive cells in each stratum of CA3 results in the three layers with the highest immunoreactivity levels, namely, stratum pyramidal, stratum molecular and stratum lucidum. The average number of cells and their deviation standard are stratum pyramidal (123.33±15.88 cells/mm^2^), stratum molecular (63±13.11 cells/mm^2^), stratum lucidum (62.67±8.08 cells/mm^2^), stratum radiatum (55.33±510.21 cells/mm^2^), stratum oriens (48±3.46 cells/mm^2^), and stratum alveus (28.67±2.52 cells/mm^2^) (Figure-8).

### Serotonin immunoreactive cells on DG

**Figure-9:**
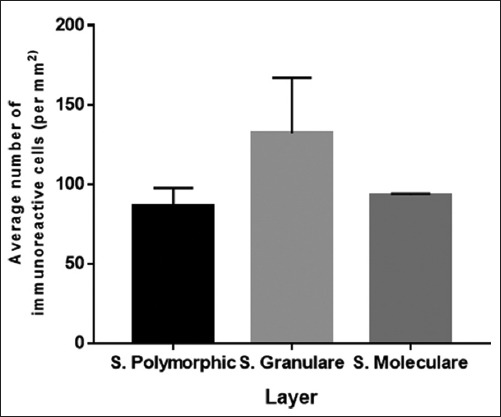
The number of immunoreactive cells from the highest to lowest in dentate gyrus start from stratum granular, stratum molecular, and stratum polymorphic.

Serotonin immunohistochemistry staining of *R. amplexicaudatus* DG area shows high immunoreactivity level in each layer (Figure-5). It is marked by cell nucleus that turned into brown on stratum molecular (Figure-5a), stratum granular (Figure-5b), and stratum polymorphic (Figure-5c). Calculation of immunoreactive cells on each stratum of DG then obtained results the three layers with the highest immunoreactivity levels, stratum granular (132±35.03 cells/mm^2^), stratum molecular (93±1 cells/mm^2^), and stratum polymorphic (86.33±11.23 cells/mm^2^) (Figure-9). From the obtained data, the most immunoreactive cells on DG area of *R. amplexicaudatus* are stratum granular.

## Discussion

Indonesia’s rainforests are home to some of the highest levels of biological diversity in the world, including bats. *R. amplexicaudatus* is one of fruit bats species in Indonesia and classified in suborder Megachiroptera. This fruit bat is nocturnal animal and commonly active in the night. *R. amplexicaudatu*s is a frugivore. It has good visualization ability which promoted both sensory and behavioral adaptations that allow them to find ripe the fruit faster. Hippocampus is central part of learning and memory; it is located in the temporal lobe of the cerebrum [7]. Our presence study with the hematoxylin-eosin staining describes the hippocampus consists of several areas; there are hippocampus proper or CA, DG, and septa subiculum (Figure-1a). This region is similar to insectivore bats, rats, and rabbit [3,7,8]. Hippocampus proper is divided into four parts based on the density level, size, and branch of axons and dendrites from pyramid cells. Regio of hippocampus proper is regio I CA1, Regio II CA2, Regio III CA3, and Regio IV CA4. Hippocampus proper has several stratum, consist of stratum alveus, stratum oriens, stratum pyramidal, stratum lucidum, stratum radiatum, and stratum molecular (Figure-1b) [7-9]. Stratum alveus is located in the dorsal part, composed of pyramidal cells axons. Stratum oriens is the second layer which consists of basal dendrites of pyramidal cells and interneuron cells. Stratum pyramidal composed of pyramidal cell bodies. Stratum lucidum contains mossy cells; meanwhile, stratum radiatum is built by the end of mossy cells. Stratum molecular which located on the ventral area contains neuron terminal from cortex entorhinal; this stratum also consists of interneuron [9].

DG is part of the hippocampus, which has a shape that resembles letter U or V (Figure-1b). DG has several stratum; these are stratum molecular, stratum granular, and stratum polymorphic (Figure-1b and 2a) [8,9]. Stratum molecular is located on the ventral part of DG, this layer composed of apical dendrites from granular cells and perforant path. This stratum is innervated by neuron plexus, and it is found condensed serotonergic fibers on infrapyramidal blade on the ventral molecular layer. Stratum granular consists of granular cells; this stratum receives serotonergic innervation by varicose fiber [8,10]. Polymorphic stratum is the innermost layer of DG; this stratum also receives serotonergic innervation. The axon from granular cells will form a synapse with the mossy cells of stratum polymorphic. Mossy cells play a role in projecting the impulse back to the granular cells (Figure-2b-d) [8,11]. The structure of the layer is similar to DG of rats and rabbit, which also consist of stratum molecular, stratum granular, and stratum polymorphic [7].

Hippocampus is closely related to learning and memory activities. It plays an important role in spatial memory, which conceptualized as subtype of episodic memory due to the retention of information in temporal-spatial. The hippocampus also contributes to long-term memory which includes episodic, contextual, spatial, and social memory [12,13]. Serotonin (5-HT) is a monoamine neurotransmitter which plays a role in the central nervous system. The role of serotonin in the central nervous system is related to mood, behavior, and learning-memory activity [14]. Learning and memory activities are centered in the hippocampus. It starts from nerve cells originating from entorhinal cortex to the DG through perforant path. Granular cells of DG conduct the impulses through axons in the pyramidal cells in CA3. Pyramidal cells in CA3 will continue the input to CA1 area through Schaffer collateral which then proceeds to subiculum. Those projections from CA1 and subiculum will be returned to entorhinal cortex [9].

In the present study, we found that the immunoreactivity levels of serotonin in CA3 area are very high (Figures-4 and 6). The highest of serotonin distribution is on CA3 area; propose because almost all subtypes of serotonin receptor are found in CA3. The presence of these receptors affects immunoreactivity level on each stratum. Dominant receptors on this stratum are 5-HT_1F_, 5-HT_2A_, 5-HT_2C_, and 5-HT_3_ and another receptor which distributed on CA3 area [15]. Serotonin immunoreactive cells are mainly found in stratum pyramidal of CA3, which has the highest cell density (Figures-4 and 6). Serotonin expression on CA3 area mainly in pyramidal cells is thought to be related to the regulation of hippocampal function and its function in retrieving previous memory and plays a role in spatial memory related to navigation.

CA1 area in learning and memory activities serves to adjust the input that received from CA3 area and afferent input from entorhinal cortex of CA1 pyramidal cells. The immunoreactivity levels of CA1 area are not as much as CA3 area (Figures-3, 6 and 7). It occurs because the cell density and the number of the receptor such as 5-HT_1F_, 5-HT_2A_, 5-HT_2C_, and 5-HT_3_ on CA1 area are not as dense and not as much as the receptors in CA3 area [4].

DG area in the hippocampus has a role in neurogenesis process. Receptors which contributes important role are 5-HT_1A_ receptors. These receptors are expressed by neuron raphe serotonin as autoreceptor that takes the role in regulates serotonin balance level in hippocampus. New neurons will grow to be granular cells on stratum granular to maintain hippocampus function in mediating and modulating learning and memory process [15]. Area on DG which most innervated by serotonin is stratum granular because it has a high density of neurons (Figures-6 and 9). Meanwhile, serotonin innervation on stratum molecular and polymorphic is not as much as stratum granular so that in immunohistochemical staining results will appear neurons with different levels of immunoreactivity [8]. Neurogenesis process begins with the reception of synaptic input from entorhinal cortex which will be sent to the target cell in CA3 area [4,9,16]. 5-HT transporter expression seems to be a reliable neural marker related to memory mechanisms and its alterations. Our finding describes clearly that the serotonergic nerve hampers widely in all of the parts of hippocampus. This result in line also with the previous data on the rat which is mention the serotonin is distributed on the CA, subiculum and DG of the rats [17]. From here, we can postulate that the tremendous ability in learning memory of the *R. amplexicaudatus* is promoted by the serotonin receptor. Previous research, already mentioned on the rat models that serotonin (5-HT) receptors mediate the potentiation of CA3-to-CA1 inputs thus may regulate memory formation and Schaffer collateral excitation [4,18]. Moreover, 5-HT represents important pharmacological targets for cognition enhancement [19,20].

## Conclusion

The serotonergic nerve is involved in the memory formation of the *R. amplexicaudatus*. In addition, the serotonin immunoreactive cells found widely in the area of hippocampus gradually from CA3, CA1, and DG.

## Authors’ Contributions

HW designed the experiments and study. VW, NDAW, GG, and GRS performed the experiments. VW, PH, and HW interpreted the data. PH and HW wrote the manuscript. All authors read and approved the final manuscript.
